# Supplementary material for: Polysubstance use among adolescents in Malaysia: Findings from the National Health and Morbidity Survey 2017
Source: PLoS One. 2021 Jan 21;16(1):e0245593. doi: 10.1371/journal.pone.0245593 (PMC7819602; doi:10.1371/journal.pone.0245593)
Supplement: Attachment — Submitted filename: 220620 Response to reviewer plos one.docx [file pone.0245593.s001.docx]

Wan Shakira Rodzlan Hasani

Institute for Public Health,

National Institutes of Health,

Ministry of Health Malaysia

No. 1, Jalan Setia Murni U13/52,

Seksyen U13, Setia Alam,

40170 Shah Alam, Selangor

Malaysia

25^th^ Jun 2020

Dear Editor-in-chief,

PLOS one

Thank you very much for your comments. We are thankful to the editors and reviewers for their time and insightful comments on our manuscript. We have amended their comments and suggestions and hope to submit a revised version of the manuscript for further consideration in the journal.

For your information, we changed the title to from **“single and Polysubstance use among adolescents in Malaysia: Finding from the National Health and Morbidity Survey 2017”** to **“Polysubstance use among adolescents in Malaysia: Finding from the National Health and Morbidity Survey 2017”.** Below, we also include a point-by-point feedback explaining how we have addressed each of the editors or reviewers’ comments. We look forward to the outcome of your assessment.

On behalf of the co-authors

Sincerely,

Wan Shakira Rodzlan Hasani

Researcher

Institute for Public Health,

National Institute of Health,

Ministry of Health Malaysia

Response to academic editor comments

| No | Comment | Response |
| --- | --- | --- |
| 1 | Please ensure that your manuscript meets PLOS ONE's style requirements, including those for file naming. | Done |
| 2 | Please provide additional details regarding participant consent. In the ethics statement in the Methods and online submission information, please ensure that you have specified (1) whether consent was suitably informed and (2) what type you obtained (for instance, written or verbal). Since your study included minors under age 18, state whether you obtained consent from parents or guardians. If the need for consent was waived by the ethics committee, please include this information. | Thank you for your suggestion. We added this statement in Method under Ethical approval “Prior to the survey, several meetings with person in charge at selected schools were conducted to inform them about the survey and brief about the parents’ consent form. The consent forms were distributed a week prior the survey. Students and their parents’ consent forms were collected just prior to the actual day of survey. Student who did not receive parental or guardian consent or they themselves refused to participate were considered as non-response of eligible participants in this survey*”* |
| 3 | We noticed you have some minor occurrence(s) of overlapping text with the following previous publication(s), which needs to be addressed: https://doi.org/10.1186/1471-2458-14-1044 https://doi.org/10.1177%2F1010539519865053 https://doi.org/10.1371/journal.pone.0207472  In your revision ensure you cite all your sources (including your own works), and quote or rephrase any duplicated text outside the Methods section. Further consideration is dependent on these concerns being addressed | All the overlapping text was changed accordingly as highlighted in track changes file.  Thank you for your comment. |
| 4 | In your Data Availability statement, you have not specified where the minimal data set underlying the results described in your manuscript can be found. PLOS defines a study's minimal data set as the underlying data used to reach the conclusions drawn in the manuscript and any additional data required to replicate the reported study findings in their entirety. All PLOS journals require that the minimal data set be made fully available. For more information about our data policy, please see http://journals.plos.org/plosone/s/data-availability.  Upon re-submitting your revised manuscript, please upload your study’s minimal underlying data set as either Supporting Information files or to a stable, public repository and include the relevant URLs, DOIs, or accession numbers within your revised cover letter. For a list of acceptable repositories, please see http://journals.plos.org/plosone/s/data-availability#loc-recommended-repositories. Any potentially identifying patient information must be fully anonymized.  Important: If there are ethical or legal restrictions to sharing your data publicly, please explain these restrictions in detail. Please see our guidelines for more information on what we consider unacceptable restrictions to publicly sharing data: http://journals.plos.org/plosone/s/data-availability#loc-unacceptable-data-access-restrictions. Note that it is not acceptable for the authors to be the sole named individuals responsible for ensuring data access. | The National Institute of Health, Ministry of Health Malaysia (data ethics committee) has placed restriction on sharing the full dataset due to cases involving researchers manipulating the data. Interested researchers will need to send a formal letter/email to the Director General of Health Malaysia, together with the data request form and proposal, available at (http://iku.moh.gov.my/index.php/research-eng/data-request-eng). The proposal will be reviewed by the Data Repository team from Biostatistics Sector, National Institute of Health Malaysia to ensure no duplication with other projects that have used the NHMS 2017 data. The data request flow chart is available on the website and can be accessed here (http://iku.moh.gov.my/images/IKU/Document/Form/FlowChartforIPHDataApplication.pdf). The authors also confirm they did not have any special access privileges that others would not have for the NHMS 2017 data. |

Response to reviewer’s 1 comments

| No | Comment | Response |
| --- | --- | --- |
| 1 | Introduction |  |
|  | What is the impacts of polysubstance use and why is such study important in prevention and treatment of substance users? The information would be mentioned in introduction to help readers to better understand the importance of your study. In addition, on what basis did you choose those variables/risk factors? Also, in your introduction part, there was a very little usage of literature conducting in Malaysia and the neighboring countries on the similar event. | Thank you for your suggestion. We added the impacts of polysubstance use, factor related to substance use and prevalence of combination substance in Malaysian and neighboring countries (South-east Asia). Almost all paragraphs in introduction part were changed accordingly in order to help reader understand the important of this study. |
| 2 | Method |  |
|  | The authors have not discussed how they built the multivariate regression analysis model they are referring in Table 3 and 4 is not enough. In page 13, you described “A final model was obtained which included all the factors that were significantly associated at the level of p <0.05.” you need to describe in detail. For example, table 3 presents crude odds ratios using univariate multinomial logistic regression. In cases of some variables, such as parental or guardian never/rarely respect for privacy, anxiety, and stress, showed statistically significances in table 4. but they were not included in the multivariate model. How did you build the multivariate regression analysis? | Thank you for your suggestion. This statement (below) on how the multivariate regression model was build were added under method section.  “Univariable analysis was carried out in this study by testing all the 15 potential predictor variables in order to screen for important independent variables. Considering the variables with *p-*values <0.25 from univariable analysis and important variables based on biological plausibility, all those 15 variables were included in the preliminary final model (variable selection). The variable selection is the process of “reducing the model” in order to get the best fit model by including all the candidate variable in the model, and repeatedly removing the variables with the highest non-significant p value until the model contains only significant terms. Hence, the final model was created based on ten variables that were significantly associated at the level of p <0.05 during final steps of variables selection. Those variables were age, sex, and ethnicity, marital status of parents, truancy, peer support, parent/guardian bonding, depression, close friend and being bullied” |
| 3 | Results and Discussion |  |
|  | Polysubstance use is defined as the use of more than one non-prescribed licit or illicit substance either concurrently or simultaneously. As it is difficult to understand the table 2, please revise the table readable.  In addition, the estimate in page 13 and 14 (5.1%) is different from that in page 17 (0.5%). I may assume that 0.5% is the prevalence of those who are concurrent use of three substances (alcohol, tobacco and illicit drug)? Please check and clarify the definition respectively. | Thank you for your suggestion.  For Table 2 – Revision done  5.1% is the correct value. We removed the value 0.5% in page 17. Thank you for your comment. |
| 4 | Discussion |  |
|  | The results and discussion are lacking the nuts and bolts to would make the paper relevant to policy and practice.    In addition, in Table 2, other ethnicity compared to Malay, such as Chinese, Indian and others had higher odds of being polysubstance user. In particular, Indians and other ethnicities had much higher odds. However, the authors only mentioned Chinese and others and further failed to explain why it happened among Indians and others rather than Malay.  The authors mentioned that “the current study is the first study to examine self-reported substance use among a nationally representative sample of adolescents to better estimate the prevalence of polysubstance use among Malaysian adolescents.” To my knowledge, there are some studies on polysubstance use, although some evaluated concurrent use of alcohol and tobacco and not include illicit drug use using GSHS. So you may think of what your study can add in the literature. | Thank you for opinion. Almost all paragraphs under discussion part were changed accordingly in order to make this paper relevant to policy and practice. The recommendation specific to policy & practice were added under conclusion.  Majority of other ethnicities were Bumiputeras Sabah & Sarawak. Thus the word “other” was changed to “Other Bumiputeras”. The explanation why ethnic groups other than Malay more likely became polysusbtance user were explained details in discussion.  Thanks you for your comment. The statement “the current study is the first study to examine self-reported substance use among a nationally representative sample of adolescents to better estimate the prevalence of polysubstance use among Malaysian adolescents” was removed. |
| 5 | Presentations of tables |  |
|  | I am troubled by table presentation style in Table 3 and 4. For example, in table 3, the estimates of single substance use are not in the same row. Need to revise table presentations properly in table 3 and 4.  Table 3: It would be better if you change some labels. Polysubstance use of -> Polysubstance use (delete “of”) Total -> Any substance use  Add one category of “Alcohol and tobacco and illicit drug” and the estimate. | Thank you for your suggestion.  Done editing Table 3 & 4.  Done editing label. Thank you for your suggestion.  Analysis for use all three substance (alcohol+ tobacco + drug) was done and added to Table 3. Thank you for the suggestion. |
| 6 | Reference format |  |
|  | Please check the appropriate reference format of PlOS One and revise accordingly. | Revision done based on PLOS one format. Thank you for your comment. |
