# Supplementary material for: Polysubstance use among adolescents in Malaysia: Findings from the National Health and Morbidity Survey 2017
Source: PLoS One. 2021 Jan 21;16(1):e0245593. doi: 10.1371/journal.pone.0245593 (PMC7819602; doi:10.1371/journal.pone.0245593)
Supplement: Attachment — Submitted filename: 201120 Response to reviewer.docx [file pone.0245593.s002.docx]

**Response to academic editor comments**

1. **Additional Editor Comments (if provided):**

   As per Reviewer 2's comment, this manuscript requires significant English language editing to enhance readability.
   Some additional information in the Methods section as to how consent for participation in the survey was sought and received would be helpful, as well as the duration of the survey. Was it undertaken during school time, or outside of school hours? Though the authors do note that the methods are reported elsewhere, the key methodological details should still be reported in this manuscript.

**Feedback: Thank you for your comments. The English language editing was done accordingly. The statement regarding consent for participants already stated in the methodology under ethical approval as below;**

**“Prior to the survey, several meetings with person in charge at selected schools were conducted to inform them about the survey and brief about the parents’ consent form. The consent forms were distributed a week prior the survey. Students and their parents’ consent forms were collected just prior to the actual day of survey. Student who did not receive parental or guardian consent or they themselves refused to participate were considered as non-response of eligible participants in this survey”.**

**The duration of the survey and field implementation was added in the methodology as below:**

**“Prior to the survey, the data collectors were trained during 1-week workshop. The data collection was conducted from March until May 2017. A field supervisor was assigned for each state to supervise the survey activities including arrangement the slot for survey with the school. The survey was conducted either in the classroom or school hall during school time and it took about 45 minutes to 1 hour to complete the survey. The answered OMR form was put in the envelope by each student to maintain confidentiality. ”**

**Response to reviewer comments**

2. **Review Comments to the Author**

   Reviewer #1: Thanks for the revision.

   First, Introduction: the authors made a revision. Not agree to add the sentences about patterns of polysubstance use in the USA in the first paragraph of the introduction; “In the United States, the most frequently paired of polysubstance use …….both alone and in combination.” you may move to discussion part to discuss the polysubstance use patterns among countries.

**Feedback: Thank you for your suggestion. We moved this statement to discussion.**

Second, the authors added the new variables of "all three substance use(alcohol+ tobacco + drug)." It is surprising that prevalence of alcohol+ tobacco + drug is higher than that of the other polysubstance user group. For example, the prevalence of alcohol+ tobacco + drug (2.2%) is higher compared to that of tobacco and alcohol use (2.0%).

i) why is the prevalence of alcohol+ tobacco + drug higher than that of tobacco and alcohol use (2.0%)? As illicit substance is not common, it seems interesting and also doubtable. Please discuss in discussion part.

**Feedback:** **Thank you for your comment. We agree with your point. After discuss with other co-authors, we re-analyse the prevalence of combination two substances regardless of the 3^rd^ substance use (Refer to Table 3). Previously, we exclude the third substance on combination of two substance (example combination of alcohol and tobacco excluded the drug user, combination of drug and alcohol excluded tobacco user).**

**The illustration circle of combination substance use (in the previous analysis) as below;**

**Drug**

0.5%

0.4%

11.2%

**Tobacco**

2.2%

0.4%

2.0%

5.6%

**Alcohol**

**After re-analyse, the prevalence of combination three substances (2.2%) is lower than combination of any two substances. The illustration as below**

**Drug**

**Tobacco**

2.7%

**Alcohol**

**Tobacco**

4.2%

**Alcohol**

**Drug**

2.5%

**We agree that, the current result for combination of substance is more meaningful and will interpret the result as current analysis.**

ii) in the first paragraph of the discussion, the authors described “Overall, this study showed alcohol and tobacco were the most common pair of substance use.” please clarify the sentences and the estimates in Table 3.

**Feedback: After re-analyse for Table 3, the combination of alcohol and tobacco use shows the highest prevalence as compare to combination of other substance. Thus this statement (“Overall, this study showed alcohol and tobacco were the most common pair of substance use”) will be maintain.**

Lastly, regarding the reference formatting, the references should be revised correctly. In particular, the name list of the authors were not well-formatted.

**Feedback: Thank you for your comment. The references was revised accordingly. We change to PLoS reference style using EndNote.**

3. Reviewer #2:

This article examines the prevalence of and associated factors related to single-use and polysubstance use among adolescents in Malaysia. I would like to point out that I am reviewing a revised draft of the manuscript (I did not review the initial draft); thus, my comments are relatively minor since the authors seem to have addressed many of the previous reviewers’ concerns. Overall, the subject matter is of great public health importance and the authors have attempted to present a comprehensive profile of juvenile substance use in Malaysia.

1. The introduction, while succinct, can benefit from a slightly more enhanced discussion of the topic at hand. Specifically, the following can be included:
a. Importance of identifying and/or preventing polysubstance use at an early stage.
b. Ease of access to alcohol and drugs within the context of Malaysia, that can explain the rising rates of polysubstance use.

**Feedback: Thank you for your suggestion. We revised the introduction and added the statement as suggested.**

2. I’m a little concerned about the self-reported nature of the truancy variable. Was it not possible to retrieve this information from classroom records? Similarly, the use of single items for peer support, parental bonding and parental respect for privacy, is short-sighted and unreliable.

**Feedback: Thank you for your comment. As described in method section, this study applied self-administered method for data collection using validated questionnaire (GSHS) from WHO. We followed all the protocols and guideline from WHO for GSHS. Brief information regarding GSHS and link (URL) for GSHS as below;**

**“The Global School-based Student Health Survey (GSHS) was developed by the World Health Organization (WHO) and the Centers for Disease Control and Prevention (CDC). It has been widely use by more than 100 countries, which enable international comparisons of findings.**

**The GSHS is a school-based survey conducted primarily among students aged 13–17 years. The GSHS uses a standardized scientific sample selection process; common school-based methodology; and core questionnaire modules, core-expanded questions, and country-specific questions that are combined to form a self-administered questionnaire that can be administered during one regular class period.**

**The 10 core questionnaire modules address the leading causes of morbidity and mortality among children and adults worldwide which were 1) Alcohol use, 2)Dietary behaviors, 3)Drug use, 4) Hygiene, 5) Mental health, 6) Physical activity, 7) Protective factors, 8) Sexual behaviors that contribute to HIV infection, other sexually-transmitted infections, and unintended pregnancy , 9)Tobacco use, 10) Violence and unintentional injury”**

[**https://www.cdc.gov/gshs/background/index.htm**](https://www.cdc.gov/gshs/background/index.htm)

[**https://www.cdc.gov/gshs/pdf/GSHSOVerview.pdf**](https://www.cdc.gov/gshs/pdf/GSHSOVerview.pdf)

**In the other hand, this study involved 212 selected schools with 27, 497 sample students collected in year 2017. We are unable to retrieve the information on classroom report for truancy as we did not collect that information during data collection.**

3. The writing is poor and idiosyncratic. There are multiple instances of sentences that are poorly worded and confusing to read. For example, “polysubstance use between the male and female” sounds odd. This sentence should be reworded as “polysubstance use between males and females. Consider this other statement “For example, Indigenous people (Sabah and Sarawak) is required to drink alcohol during harvest festival and social gathering [43] but Malays where mostly are Muslims are prohibited from drink alcohol according to Malaysia’s sharia law.” I realize that there is likely a language barrier and hence I would encourage the authors to seek the assistance of a copy-writer to make the necessary corrections.

**Feedback: Thank you for your comment and suggestion. The sentences were rephrased and editorial correction was done and revised accordingly.**

4. One of the most interesting findings that the authors gloss over is the that younger adolescents were more likely to engage in single substance use. How do the authors explain this result? As I mentioned in my first comment, it would be useful to understand the socio-cultural context that either constrains or emboldens substance use at a young age. This would allow for policy or structural changes to address these issues.

**Feedback: Thank you for your comment. We added the explanation on the younger adolescent more likely to engage in single substance use on the discussion.**
