# Supplementary material for: Polysubstance use among adolescents in Malaysia: Findings from the National Health and Morbidity Survey 2017
Source: PLoS One. 2021 Jan 21;16(1):e0245593. doi: 10.1371/journal.pone.0245593 (PMC7819602; doi:10.1371/journal.pone.0245593)
Supplement: Attachment — Submitted filename: Review comments_20201204.docx [file pone.0245593.s003.docx]

Thanks for your revision. I found the manuscript improved. I have more minor things to be revised though.

**Abstract**

“The NHMS utilised a two-stage stratified cluster sampling **~~using~~**.”

**Tables**

In table 2, the prevalence of the lifetime use of smoking tobacco is supposed to be greater than that of the current use. but in your analysis, the lifetime use (5.1%) vs the current use (6.7%). please check the data.

In Table 3, indicate in the table whether the estimates were crude or weighted values.

In Table 4, the 95% CI of the COR of Chinese need to be checked (COR=1.10 (95% CI=1.71-2.33)). The COR is supposed to be in the range of 95% CI.

**Conclusion**

Check the full name of “*INTIM camp* (*Kem Kecemerlangan Intelek Murid*)”
