# Supplementary material for: Polysubstance use among adolescents in Malaysia: Findings from the National Health and Morbidity Survey 2017
Source: PLoS One. 2021 Jan 21;16(1):e0245593. doi: 10.1371/journal.pone.0245593 (PMC7819602; doi:10.1371/journal.pone.0245593)
Supplement: Attachment — Submitted filename: 241220 Response to reviewer.docx [file pone.0245593.s004.docx]

**Response to reviewer comments**

1. Abstract

“The NHMS utilised a two-stage stratified cluster sampling ~~using~~.”

**Feedback: Thank you for your comments. The word “using” was removed from the abstract.**

2. Tables

In table 2, the prevalence of the lifetime use of smoking tobacco is supposed to be greater than that of the current use. but in your analysis, the lifetime use (5.1%) vs the current use (6.7%). please check the data.

**Feedback: Thank you for your comments. I do agree with you. After checked the analysis, I admit my mistake that I’m taking the wrong variable for lifetime use for smoked tobacco (the current figure demonstrated lifetime use for cigarette only). The value for lifetime use for smoked tobacco supposed be 21.4% (95%CI: 20.04, 22.84) for total, 34.3% (95%CI: 32.09, 36.55) for male and 8.7% (95%CI: 7.71, 9.88) for female. We really appreciate on your comment and apologies for the mistake. These values (in Table 2) were changed accordingly.**

In Table 3, indicate in the table whether the estimates were crude or weighted values.

**Feedback: Thank you for your comment and suggestion. Table 2 and 3 are weighted analysis using complex sampling design analysis. The statement on weighted analysis added on the table’s foot note**

In Table 4, the 95% CI of the COR of Chinese need to be checked (COR=1.10 (95% CI=1.71-2.33)). The COR is supposed to be in the range of 95% CI.

**Feedback: Thank you for your comments. I checked the output of the analysis. The value is wrongly entered. The COR of Chinese group should be 2.00, not 1.10. Thank you again for checking this table.**

3. Conclusion

Check the full name of “*INTIM camp* (*Kem Kecemerlangan Intelek Murid*)”

**Feedback: Thank you for your comments. We checked from AADK/NADA website, INTIM or “Kem Kecemerlangan Intelek Murid” currently known as PINTAR or “Program Intelek Asuhan Rohani”. No full abbreviation for INTIM was found. Thus we decided to remove the word “INTIM (Kem Kecemerlangan Intelek Murid)” and changed to PINTAR (*Program Intelek Asuhan Rohani*). The details on PINTAR program as link below:**

<https://www.adk.gov.my/en/prevention/drug-free-education-institution/>
